# Fee Bill

**Published:** 1889-07

**Authors:** 


### FEE BILL.

There is not much uniformity in medical fee bills as between different localities. In the country the charges are largely dependent upon the milage. The following rates are those ot the City of Atlanta, as adopted by the Atlanta Medical Society:

MEDICINE:

| | |
|---|---|
| Ordinary day visit | $2.00 |
| " night " | 4.00 |
| Detention charged for according to circumstances. | |
| Office consultation | $1.00 to $10.00 |
| Visit in consultation with another physician | 10.00 |
| Subsequent consultation visit | 5.00 |
| Opinion, involving questions of law | 25.00 |
| Post-mortem examination, involving questions of law | 50.00 |
| Ordinary post-mortem examination | 25 00 |
| Vaccination | 1.00 |

OBSTETRICS:

| | |
|---|---|
| Natural labor | 25.00 |
| Instrumental labor | 50.00 |
| Miscarriage, attendence upon | 25.00 |
| Vaginal examination | 5.00 |

DISEASES OF WOMEN:

| | |
|---|---|
| Vaginal fistulæ | 50.00 |
| Laceration of Perinæum | 50.00 |
| " " Cervix | 50 00 |
| Ovariotomy | 250.00 |

SURGERY:

| | |
|---|---|
| Administration of an anæsthetic | 5.00 |
| Opening abscess | 2.00 |
| Simple fracture of arm | 25.00 |
| " " " leg | 50.00 |
| " " " thigh | 100 00 |
| " " " jaw | 25.00 |
| " " " clavicle | 25.00 |
| " " " ribs | 25 00 |
| " " " patella | 50.00 |
| " " " fingers and toes | 15·00 |
| Compound fractures charged for according to circumstances. | |
| Dislocation of hip | 100.00 |
| " " shoulder | 25.00 |
| " " elbow | 25.00 |

SURGERY:

| | |
|---|---|
| Dislocation of wrist | 25.00 |
| " " knee | 25.00 |
| " " ankle | 25.00 |
| " " jaw | 15 00 |
| " " finger or toe | 15.00 |
| Reduction of hernia, simple | 10.00 |
| " " " strangulated | 25.00 |
| Tapping abdomen | 20 00 |
| " hydrocele | 10.00 |
| " thorax | 25.00 |
| Operation for varicocele | 25.00 |
| " " fistula in ano | 25.00 |
| " " hair lip | 75.00 |
| Introduction of sound | 2.00 |
| Treatment of stricture by gradual dilation | 50.00 |
| Internal urethrotomy | 50 00 |
| External urethrotomy | 75.00 |
| Operation for phymosis or paraphymosis | 15.00 |
| Examination of rectum | 5.00 |
| Operation for external hemorrhoids | 10.00 |
| " " internal " | 50.00 |
| " " varicose vein | 50.00 |
| Circumcision | 25.00 |
| Cutting meatus | 30.00 |
| Amputation of thigh | 100.00 |
| " " leg | 100 00 |
| " " arm | 50.00 |
| " " shoulder | 100.00 |
| " " breast | 75.00 |
| " " fingers and toes | 15.00 |
| " " penis | 75 00 |
| " " uvulva | 10 00 |
| " " tonsil | 20.00 |
| Tracheotomy | 75 00 |
| Operation for nasal and aural polypi | 20.00 |
| Passing catheter | 5.00 |
| Treatment of gonorrhœa | 10.00 |
| " " syphilis | 50.00 |
| " " chancroid | 10.00 |
| Radical operation for hydrocele | 30.00 |

The above charges to be *minimum* charges, except where the physician's knowledge of a patient's financial circumstances leads him to believe them unable to pay, when he may make such deductions as seems fit.

